# Substance P NK1 receptor in the rat corpus callosum during postnatal development

**DOI:** 10.1002/brb3.713

**Published:** 2017-05-02

**Authors:** Paolo Barbaresi, Emanuela Mensà, Guendalina Bastioli, Salvatore Amoroso

**Affiliations:** ^1^ Section of Neuroscience and Cell BiologyDepartment of Experimental and Clinical MedicineMarche Polytechnic UniversityAnconaItaly; ^2^ Department of Biomedical Sciences and Public HealthMarche Polytechnic UniversityAnconaItaly

**Keywords:** corpus callosum, immunocytochemistry, intracallosal neurons, neurokinin, tachykinin

## Abstract

**Introduction:**

The expression of substance P (SP) receptor (neurokinin 1, NK1) was studied in the rat corpus callosum (cc) from postnatal day 0 (the first 24 hr from birth, P0) to P30.

**Methods:**

We used immunocytochemistry to study the presence of intracallosal NK1‐immunopositive neurons (NK1_IP__‐n_) during cc development.

**Results:**

NK1_IP__‐n_ first appeared on P5. Their number increased significantly between P5 and P10, it remained almost constant between P10 and P15, then declined slightly until P30. The size of intracallosal NK1_IP__‐n_ increased constantly from P5 (102.3 μm^2^) to P30 (262.07 μm^2^). From P5 onward, their distribution pattern was adult‐like, that is, they were more numerous in the lateral and intermediate parts of the cc, and declined to few or none approaching the midline. At P5, intracallosal NK1_IP__‐n_ had a predominantly round cell bodies with primary dendrites of different thickness from which originated thinner secondary branches. Between P10 and P15, dendrites were longer and more thickly branched, and displayed several varicosities as well as short, thin appendages. Between P20 and P30, NK1_IP__‐n_ were qualitatively indistinguishable from those of adult animals and could be classified as bipolar (fusiform and rectangular), round–polygonal, and pyramidal (triangular–pyriform).

**Conclusions:**

Number of NK1_IP__‐n_ increase between P5 and P10, then declines, but unlike other intracallosal neurons, NK1_IP__‐n_ make up a significant population in the adult cc. These findings suggest that NK1_IP__‐n_ may be involved in the myelination of callosal axons, could play an important role in their pathfinding. Since they are also found in adult rat cc, it is likely that their role changes during lifetime.

**Abbreviations used according to the atlas of Paxinos and Watson (1982) and Zilles (1985)**
Aq: cerebral aqueduct
CA1: field CA1 of Ammon's horn
CA2: field CA2 of Ammon's horn
CA3: field CA3 of Ammon's horn
CA4: field CA4 of Ammon's horn
cc: corpus callosum
CGD: central gray, dorsal part
CGM: central gray, medial part
CPu: caudate–putamen nucleus
df: dorsal fornix
DG: dentate gyrus
dhc: dorsal hippocampal commissure
fi: fimbria of the hippocampus
gcc: genu of the corpus callosum
GP: globus pallidus
HiF: hippocampal fissure
IC: inferior colliculus
IG: indusium griseum
LSD: lateral septal nucleus, dorsal part
LV: lateral ventricle
S: subiculum
scc: splenium of the corpus callosum
3V: third ventricle

## 1 Introduction

The corpus callosum (cc), the largest fiber tract connecting the two cerebral hemispheres, is made up of axons whose cell bodies are located in layers II/III and V of the cerebral cortex (Innocenti, 1986). In the adult, the majority of callosal fibers use excitatory amino acid neurotransmitters such as glutamate and aspartate (Barbaresi, Fabri, Conti, & Manzoni, 1987). In contrast, a small contingent of fibers are immunopositive for the inhibitory transmitter γ‐aminobutyric acid (GABA, Gonchar, Johnson, & Weinberg, 1995; Fabri & Manzoni, 2004; Higo, Akashi, Sakimura, & Tamamaki, 2009) or for modulatory peptides such as cholecystokinin (CCK; Seroogy, Fallon, Loughlin, & Leslie, 1985), neuropeptide Y (NPY; Ding & Elberger, 1994, 2000; Woodhams et al., 1985), or somatostatin (SOM; Ding & Elberger, 2000).

The mature cc also contains both astrocytes and oligodedendrocytes (Innocenti, 1986), and many studies performed in different species, including humans, have described the presence of neurons.

First, Malobabić, Bogdanović, and Drekić (1984), using the Golgi method, described some multipolar neurons in the human cc, whose dendrites intermingled with callosal fibers. More recently, Riederer, Berbel, and Innocenti (2004) and Revishchin, Okhotin, Korochkin, and Pavlova (2010) have demonstrated microtubule‐associated protein 2 (MAP2) and calretinin (CR)‐positive cells in the cat and rat cc, respectively, using immunocytochemical methods. Nitric oxide (NO)‐producing neurons have been described in both the monkey and rat cc (Barbaresi, Fabri, & Mensà, 2014; Rockland & Nayyar, 2012). The rat cc also contains neurons that express neurokinin 1 receptor (NK1_R_; Barbaresi et al., 2015), the receptor with the highest affinity for substance P (SP; Harrison & Geppetti, 2001; Onaga, 2014). In the adult rat cc, NK1_R_ is expressed by the overwhelming majority of NO‐producing neurons (Barbaresi et al., 2015), which therefore release NO through the action of SP.

SP also elicits a variety of effects by activating multiple subtypes of tachykinin receptors. Such effects appear to be involved not only in synaptic transmission, but also in synaptic plasticity during development of the mammalian central nervous system (CNS); in particular, several studies suggest that NK1_R_ may play a role in the synaptic plasticity associated with morphological and CNS functional development (Jonakait, Ni, Walker, & Hart, 1991; Ni & Jonakait, 1988; Quirion & Dam, 1986).

In many cases, these neurons, known as “intracallosal neurons” (Jovanov‐Milošević, Petanjek, Petrović, Judaš, & Kostović, 2010), decrease during the postnatal period. In cats the number of MAP2‐positive intracallosal neurons drops from 570 at birth to about 200 in the adult cc. Moreover, their distribution changes with age. At first, they are found throughout the cc, whereas in the adult they are confined to the rostrum (Riederer et al., 2004). CR‐positive neurons have been detected on the ventral border of mouse cc during the early stages of postnatal development (Revishchin et al., 2010). In the human cc, intracallosal neurons are particularly numerous in the second half of gestation and in the early postnatal years, but are sporadically found in the adult brain (Jovanov‐Milošević et al., 2010). The above studies strongly suggest that the developing cc contains populations of transient neurons.

The present study was devised to gain insight into the possible involvement of SP in early postnatal cc development. To do this, an antibody against NK1_R_ (Shigemoto et al., 1993) was used to verify the presence of immunopositive intracallosal neurons in rats of different ages, from postnatal day 0 (P0) to P30 and to assess their size, morphology, and distribution during postnatal development.

## 2 Material and Methods

### 2.1 Animals

The study involved 43 Sprague Dawley albino rats of different ages whose care and handling were approved by the Animal Research Committee of Marche Polytechnic University in accordance with National Institutes of Health guidelines. All efforts were made to minimize animal suffering and to reduce the number of animals used.

### 2.2 Histological procedures

#### 2.2.1 Definition of stereotaxic levels

Animals came from three different litters and were examined at seven different ages. The day of birth (the first 24 h from birth) was considered as day 0 (P0).

Animals from each group (P0, P5, P7, P10, P15, P20, P30) was anesthetized with chloral hydrate (12% in phosphate buffer, PB; 0.1 mol/L; pH 7.4) and perfused with saline followed by 4% paraformaldehyde and 20% saturated picric acid in PB (0.1 mol/L; pH 7.4). Brains were removed and postfixed for 8–12 hr in the same fixative used for perfusion and then placed in increasing solutions of sucrose (10%, 20%, 30% in PB; at 4°C) for cryoprotection, until they sank. Each brain was cut in the sagittal plane into 60‐μm serial sections using a freezing microtome. Sections were collected in PB (0.1 mol/L; pH 7.4), mounted on subbed slides, stained with neutral red (Fluka Chemie GmbH, Buchs, Switzerland; 1% in aqueous solution), and covered by coverslip. They were analyzed by light microscopy to identify, in each age group, stereotaxic levels comparable with those of the adult. The stereotaxic levels selected were lateral (lat) 3.9, 2.9, 1.9, 0.9, 0.4. At these levels the following nuclei were easily recognizable even at P0:

3.9: fimbria (fi), hippocampus, parasubiculum (PaS), presubiculum (PrS), lamina dissecans entorhinal cortex (DsC).
2.9: fimbria (fi), hippocampus, inferior colliculus (IC), and superior thalamic radiation (str).
1.9: fimbria(fi), hippocampus, anterior pretectal area (APT).
0.9: central gray (CG), fasciculus retroflexus (fr), stria medullaris thalamus (sm).
0.4: central gray (CG), fornix (f), stria medullaris thalamus (sm).

#### 2.2.2 Immunocytochemistry

The following animals were employed for immunocytochemical procedures: P0 (*n* = 9: P0‐NK1/1‐9), P5 (*n* = 9: P5‐NK1/1‐9), P7 (*n* = 6: P7‐NK1/1‐6), P10 (*n* = 4: P10‐NK1/1‐4), P15 (*n* = 4: P15‐NK1/1‐4), P20 (*n* = 4: P20‐NK1/1‐4), and P30 (*n* = 4: P30‐NK1/1‐4). Rats were deeply anesthetized with chloral hydrate (12% in PB) and perfused as described above. Brains were removed and postfixed for 8–12 hr in the same fixative used for the perfusion and then placed in increasing sucrose solutions (10%, 20%, 30% in PB; at 4°C) for cryoprotection, until they sank. Most cerebral hemispheres were cut sagittally, the others were cut coronally. Brains were sliced into 60‐μm‐thick sections (four consecutive) on a freezing microtome.

A section in every four was placed in PB (0.1 mol/L; pH 7.4) and then mounted on subbed slides, stained with neutral red (1% in aqueous solution), and covered by coverslip.

Three sections per stereotaxic level were selected according to the criteria reported above (see Definition of stereotaxic levels), washed in phosphate‐buffered saline (PBS; pH 7.4, 0.1 mol/L), and placed in 1% H_2_O_2_ for 30 min, to block endogenous peroxidase. They were then rinsed three times (10 min each) in PBS and pretreated for 1 hr in 20% normal goat serum and 3% Triton X‐100 (Merck KGaA, Darmstadt, Germany). Sections were incubated with the primary antibody (NK1 antibody, 1:1000/1500; for 10–12 hr), which was generously provided by Prof. R. Shigemoto and washed again in PBS (3 × 10 min). They were then incubated in secondary biotinylated goat anti‐rabbit antibody diluted 1:100 in PBS (1 hr; bGaR, BA‐1000; Vector Laboratories, Burlingame CA, USA) washed again, and then placed in avidin–biotin complex (Vectastain, ABC kit; Vector, 1:100; Hsu, Raine, & Fanger, 1981). Sections were washed in PBS (4 × 15 min) and then reacted with 0.025% 3,3′‐diaminobenzidine tetrahydrochloride (DAB, Sigma, St. Louis, MO, USA) and 0.0008% H_2_O_2_ (Merck KGaA) in Tris buffer (pH 7.4, 0.5 mol/L). Finally, sections were mounted on subbed slides, dehydrated in graded series of alcohol, cleared in xylene, and then covered by coverslip with Eukitt (O. Kindler‐GmbH, Freiburg, Germany). Moreover, a section related to all other stereotaxic levels was regularly reacted for NK1 immunocytochemistry. Sections from P0, P5, and P7 rats were reacted together with sections from P30 animals; the overlying cerebral cortex, caudate putamen (CPu), globus pallidus (GP), and mesencephalon were used as positive controls. The pattern of NK1 immunopositive neurons (NK1_IP‐n_) in these CNS regions was consistent with previous studies (Barbaresi, 1998; Barbaresi et al., 2015; Horie et al., 2000; Kaneko, Shigemoto, Nakanishi, & Mizuno, 1994; Mensà, 2013; Shigemoto et al., 1993).

### 2.3 Characterization of the NK1 antibody

The NK1_R_ antibody was made in rabbit against a peptide corresponding to amino acid residues 349–407 of rat SP receptor; its specificity has been verified by preabsorption with *trp E*‐SPR fusion protein, which abolished all staining (see Figure 2c of Shigemoto et al., 1993). The antibody has successfully been used in previous studies of the distribution of NK1_IP‐n_ in the striatum (Shigemoto et al., 1993), throughout the CNS (Nakaya, Kaneko, Shigemoto, Nakanishi, & Mizuno, 1994), in the rat cerebral cortex (Kaneko et al., 1994), periaqueductal gray matter (Barbaresi, 1998), and cc (Barbaresi et al., 2015).

### 2.4 Distribution and quantification (number, size)

#### 2.4.1 Distribution

The distribution of NK1_IP‐n_ in the cc was drawn using a camera lucida attached to a Leitz Orthoplan microscope equipped with a 10× objective (Leica, Wetzlar, Germany). Callosal boundaries were obtained by comparing the sections counterstained with neutral red with those reported in the atlas of Paxinos and Watson (1982) and Zilles (1985). Three comparable lateral stereotaxic levels (lat 3.9, lat 1.9–2.00, lat 0.6–0.4) were selected to study the distribution of intracallosal NK1_IP‐n_ in each of the following age: P5, P10, P15, and P30. All cc profiles were digitized with the Epson Perfection 3170 scanner (300 dpi resolution) connected to the Power Macintosh G5. Photographic montages were created in Adobe Photoshop CS4 Extended (Version 11.0; Adobe System, Inc., CA, USA).

#### 2.4.2 Count (number)

Three cases from P0, P5, P10, P15, P20, and P30 rats were used. Counts were performed by pooling together data from three adjacent sagittal sections at five stereotaxic levels (or stereotaxic levels comparable with those of the adult): lat 3.9, lat 2.9, lat 1.9, lat 0.9, and lat 0.4. Forty‐five sections per age group were used for counting the number of NK1_IP‐n_, overall 270 sections. Student's *t* test was used for statistical comparisons. *p *≤ .05 (*) was considered statistically significant.

#### 2.4.3 Soma size

Intracallosal NK1_IP‐n_ were randomly selected for soma size analysis according to the following criteria: (1) neurons must be intensely labeled and must show a clearly distinguishable morphology; (2) cell bodies must be located centrally within the 60‐μm section depth in order to minimize the cutting of dendritic branches near the section surface; (3) dendrites must not be overly obscured by other heavily stained processes from nearby cells; (4) dendritic trees must not show discontinuity with their cell bodies. For each age group, soma size was obtained by pooling data from three different rats.

The outlines of all somata were drawn with a camera lucida attached to a Leitz Orthoplan microscope equipped with a 100× objective (Leica). Soma profiles were digitized with the Epson Perfection 3170 scanner (300 dpi resolution) connected to the Power Macintosh G5 (Apple Italia, Srl; Milano, Italy). The size of NK1_IP_ neurons, measured as square microns, was calculated using the NIH Image program (Rasband & Bright, 1995).

### 2.5 Photomicrographs

Photomicrographs of NK1_IP‐n_ were acquired using an Eclipse E 600 microscope (Nikon‐Italia, Firenze, Italy) provided with a DS‐Vi1 color camera (Nikon Instruments, Europe BV, Kingston, Surrey, UK). Photographic montages of intracallosal neurons were created in Adobe Photoshop CS4 Extended (Version 11.0; Adobe System, Inc.); all images were cropped to appropriate size and adjusted only for brightness and contrast.

## 3 Results

### 3.1 Morphology, size, and distribution of intracallosal NK1_IP‐n_

#### 3.1.1 Postnatal day 0 (P0)

The immunocytochemical procedure yielded excellent Golgi‐like staining of neurons and their processes at all ages studied.

**Figure 1:**
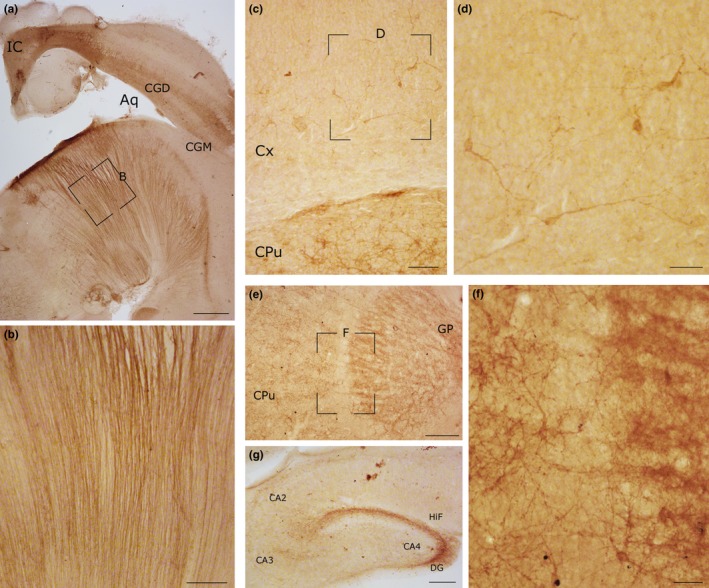
Photomicrographs showing NK1 immunoreactivity in different regions of the rat CNS at P0. (a) A dense plexus of NK1_IP_ fibers in the medulla oblongata. The framed region is enlarged in b. (b) Labeled fibers, probably immature glia processes. (c) Numerous NK1_IP__‐n_ in the CPu and cerebral cortex. The framed region, enlarged in d, shows NK1_IP__‐n_ in the subcortical plate of the cerebral cortex. (d) Several NK1_IP__‐n_ with different morphologies in the cerebral cortex. (e) NK1 labeling in the CPu and GP. The framed region, enlarged in f, shows numerous NK1_IP_ processes and neurons. (g) Immunoreactivity in the rat hippocampus. Calibration bars: a, 500 μm; b, c, and g, 100 μm; d and f, 50 μm; e, 250 μm

Intracallosal NK1_IP‐n_ were not detected at P0, but a dense plexus of intensely labeled fibers (probably glial processes; Horie et al., 2000) extending from the base of the medulla to the floor of the fourth ventricle was found in the same sections, at the most medial levels of the medulla oblongata (Figure 1a,b). Labeled neurons and dendrites were also found in CPu and GP (Figure 1c,e–f), hippocampus (Figure 1g), and the subcortical plate of the cerebral cortex (Figure 1c,d).

#### 3.1.2 Postnatal day 0 (P0) to postnatal day 5 (P5)

**Figure 2:**
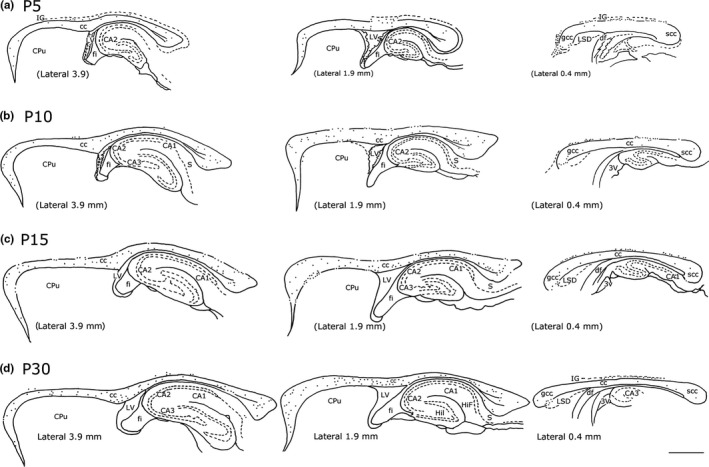
Distribution of NK1_IP__‐n_ present in the rat cc at different ages. a: P5; b: P10; c: P15; d: P30. Three different lateromedial levels/time point are shown. Numbers in the bottom left in D indicate lateromedial levels according to the atlas of Paxinos and Watson (1982). Numbers in parentheses in the bottom left corner in a, b, c indicate a lateromedial level comparable to that of the adult. Calibration bar: 1 mm

**Figure 3:**
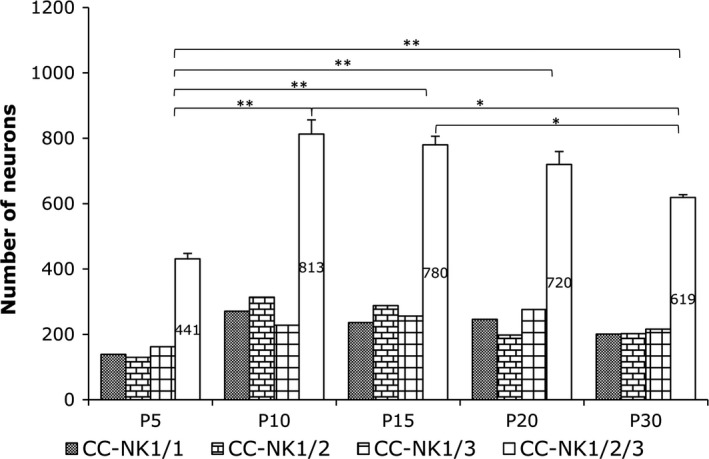
Age‐related change in the number of intracallosal NK_IP__‐n_. Counts were performed on pooled data from three rats per time point (45 sections/time point, 270 sections overall). Data were subjected to Student's *t* test. The difference between P5 and P10 was significant (**p ≤ .01)

**Figure 4:**
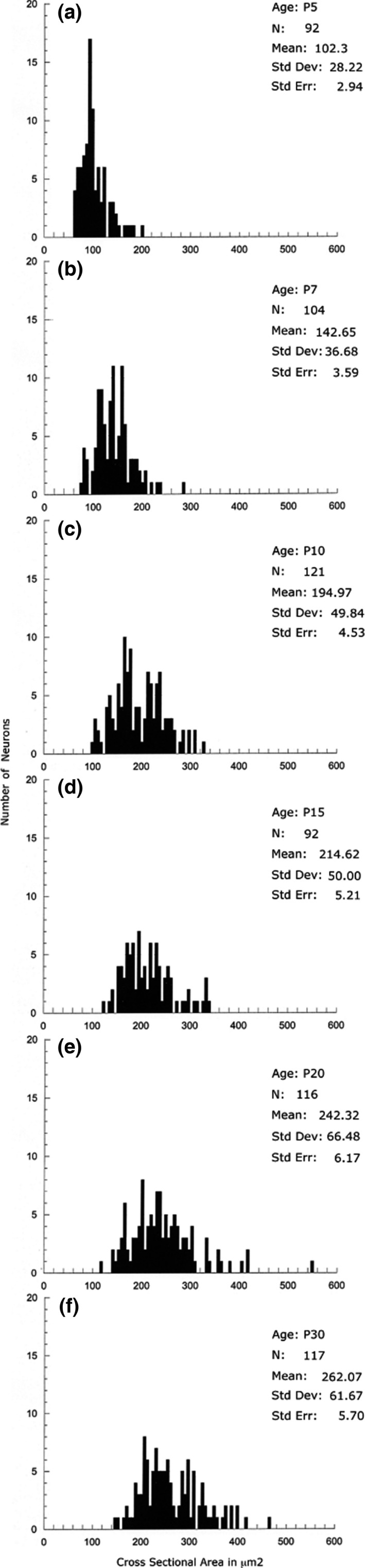
Histograms showing the size increase of intracallosal NK1_IP__‐n_ at different postnatal ages (a: P5; b: P7: c: P10; d: P15; e: P20; f: P30). Each histogram shows pooled data from three rats

**Figure 5:**
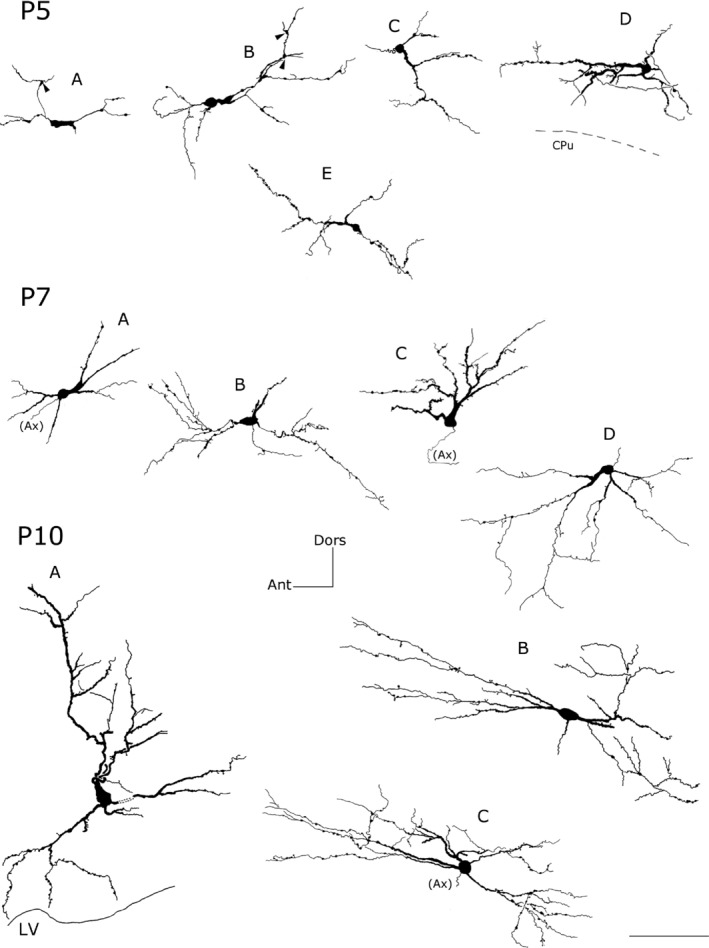
Camera lucida reconstruction of intracallosal NK1_IP__‐n_ at three different postnatal ages (P5‐P7‐P10). P5‐A: a morphologically immature NK1_IP__‐n_; P5‐B‐E: round neurons; P7‐A, ‐D: round neurons; P7‐B: bipolar neuron; P7‐C, P10‐A: triangular neurons; P10‐B: bipolar neuron P10‐C: round (polygonal) neuron. Arrowheads in P5‐A and P5‐B indicate preterminal growth buds at branching points. Calibration bar: 100 μm. Dors, dorsal; Ant, anterior; Ax, axon

**Figure 6:**
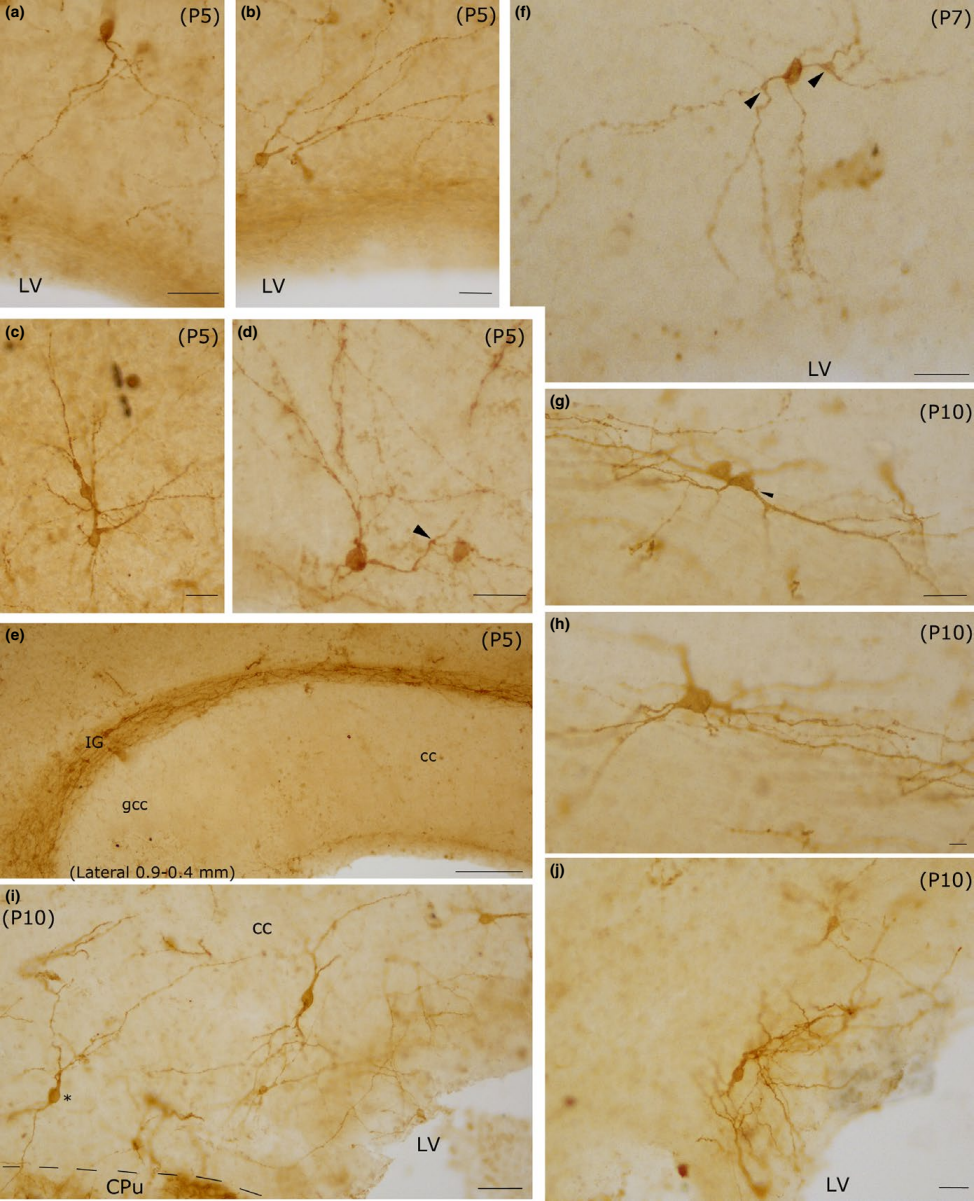
Photomicrographs of intracallosal NK1_IP__‐n_ at three postnatal ages (P5‐P7‐P10). a–e: P5; f: P7; g–j: P10. a–d: Four intracallosal NK1_IP__‐n_ showing different morphologies. (a) An ovoid NK1_IP__‐n_ with a thick principal dendrite directed toward the ependymal cc region. (b and d) Two round NK1_IP__‐n_ close to the ependymal cc region, whose dendrites are directed toward the dorsal, posterior and anterior cc regions. (c) Two adjacent NK1_IP__‐n_ in the middle of the cc. (e) Medial cc region, probably between lateral at 0.9 and 0.4 mm (comparable with those of the adult): no neurons are found at this cc level. Several NK1_IP__‐n_ are visible over the cc, in the IG. (f) An ovoid NK1_IP__‐n_ with dendrites directed in all directions, including the ependymal cc region. Arrowhead: a growth bud. (g) Polygonal NK1_IP__‐n_. (i) Several intracallosal NK1_IP__‐n_. A neuron (asterisk) sends its dendrites into the CPu. (j) A bipolar NK1_IP__‐n_ close to the ependymal region of the cc. Arrowheads in d and f indicate growth buds at branching points. Calibration bars: 25 μm in a–d, f, g, i, j; 100 μm in e; 10 μm in h

This stage was characterized by the appearance of a large number of intracallosal NK1_IP‐n_ (Figures 2 and 3) whose soma size was 102.30 μm^2^ (Figure 4a). Some exhibited typical feature of immature neurons (Figure 5, P5‐A, ‐B). The soma was irregular and gave rise to thin primary dendrites (Figure 5, P5‐A); in other cases, NK1_IP‐n_ perikarya gave rise to a thick principal dendrite from which originated thinner secondary branches (Figure 5, P5‐B‐E). The more mature neurons displayed a more regular morphology of the soma, which was essentially round (Figure 6b–d). Often, varicose swellings were observed along the length of dendrites (Figures 5 and 6). Some dendrites could be followed into the ependymal cc region (Figure 6a). A typical feature of growing dendrites were terminal swellings, which were interpreted as growth cones, sometimes bearing filopodia and preterminal growth buds at branching points (Figures 5, P5‐A, ‐B, P5‐E, and 6d). Dendrites give rise to sparse appendages of varying length, the shorter ones having the appearance of spines (Figure 5).

**Figure 7:**
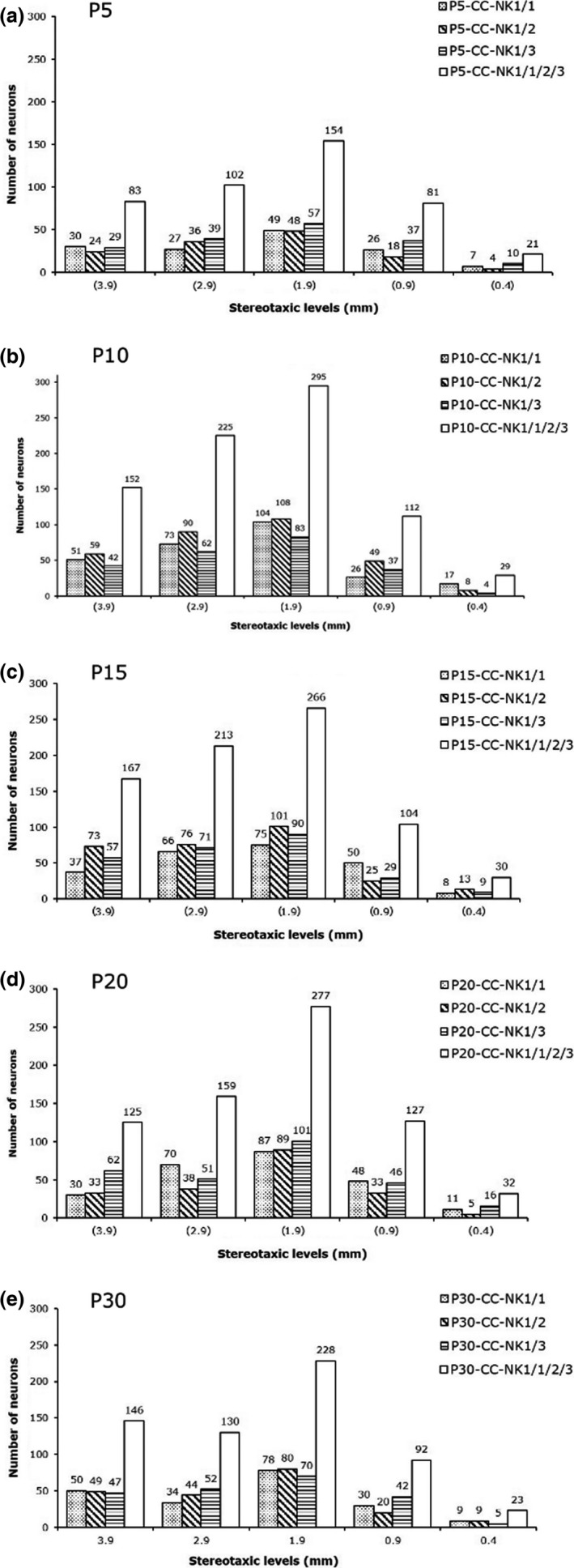
Number of NK1_IP__‐n_ detected on different stereotaxic planes from lateral (lat 3.9) to medial (lat 0.4). Five different lateromedial levels/time point are shown. Numbers in parentheses in a, b, c, d indicate lateromedial levels comparable with those of the adult. Stereotaxic levels in d (P30) indicate lateromedial levels according to the atlas of Paxinos and Watson (1982). Three rats per age (CC‐Nk1/1; CC‐NK1/2; CC‐NK1/3) are shown. Data for each rat come from pooling three adjacent sections

At P5, the distribution of intracallosal NK1_IP‐n_ was already adult‐like (Figures 2a and 7a). They were found along the whole rostrocaudal extension of the cc. They were more numerous at the lateral and intermediate levels of the rat cc (comparable with those of the adult: about lateral 3.9–2.9 and 1.9, Paxinos & Watson, 1982; Figures 2a and 7a), whereas at the midline levels (comparable with those of the adult: about lateral 0.9–0.4; Paxinos & Watson, 1982; Figures 2a and 7a) their number was very low. At this level, however, numerous NK1_IP‐n_ were found in an area just above the cc, probably corresponding to the indusium griseum (IG; Figures 2a and 6e) and in an area located anterior to the genu of the cc (probably tenia tecta; Figure 2a). Some NK1_IP‐n_ were also seen ventral to the genu on the border with the lateral septal nucleus.

#### 3.1.3 Postnatal day 5 (P5) to postnatal day 10 (P10)

From P5 onward, several intracallosal NK1_IP‐n_ acquired a significantly more mature appearance, which enabled their classification into at least three groups: bipolar (Figures 5, P7‐B, P10‐B, and 6f,i,j), round–polygonal (Figures 5, P7‐C, ‐D, P10‐C, and 6h), and triangular (Figures 5, P10‐A, and 6g). A striking increase in the complexity of dendrites occurs at this stage. Intracallosal NK1_IP‐n_ displayed longer and more branched dendrites with many varicosities; some preterminal growth buds were also observed at branching points (Figure 6f); short and thin appendages were often noted along their course (Figures 5 and 6). Often, the shorter processes still terminated into growth cones. The dendrites of intracallosal NK1_IP‐n_ located in the middle cc often reached the ependymal cc region (Figure 6f), where NK1_IP‐n_ were sometimes noted (Figures 5, P10‐A and 6i–j). In some cases, the dendrites could be followed into the overlying white matter. At this stage, soma size increased to 142.65 μm^2^ at P7 and to 194.97 μm^2^ at P10 (Figure 4b,c). In addition to the size increase, the number of intracallosal NK1_IP‐n_ also exhibited a considerable and significant increase (Figure 3; **p < .01 by Student's *t* test). As in the previous stage, NK1_IP‐n_ were detected along the rostrocaudal cc extension, with differences in their lateromedial distribution. They were more numerous at the lateral and intermediate levels and rare at the midline levels (comparable levels with those of the adult about: lat 0.9–0.4; Paxinos & Watson, 1982; Figures 2b and 7b). Medially, numerous NK1_IP‐n_ were observed just above the cc in a zone corresponding to the adult IG; their dendrites formed a long, narrow network parallel to the longitudinal cc axis and sometimes entered into the cc.

#### 3.1.4 Postnatal day 10 (P10) to postnatal day 15 (P15)

**Figure 8:**
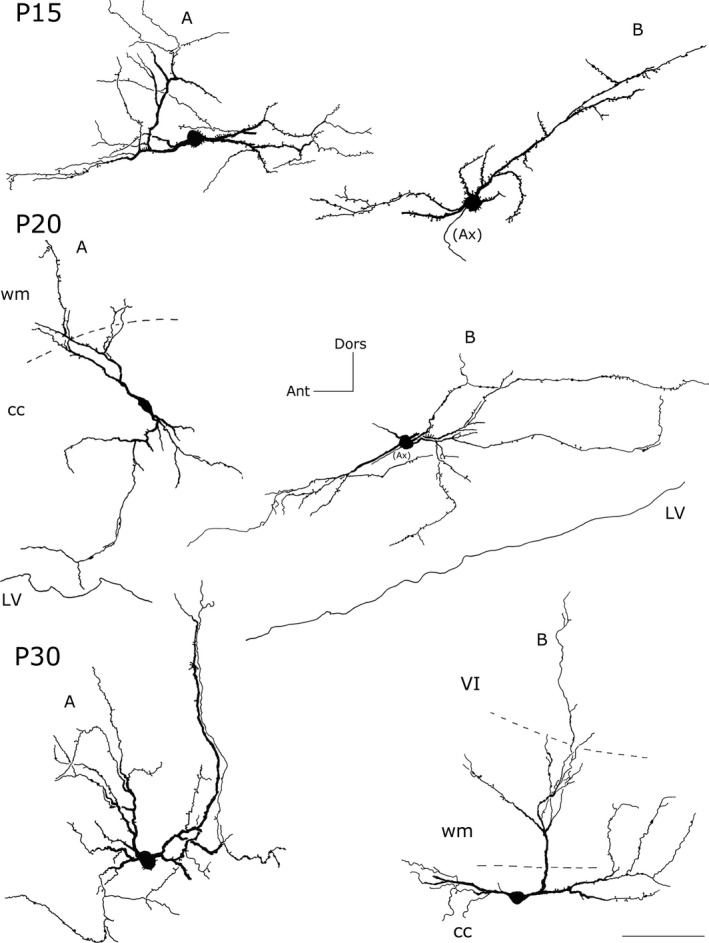
Camera lucida reconstruction of intracallosal NK1_IP__‐n_ at three different postnatal ages (P15‐P20‐P30). P15‐A, ‐B, P20‐B: round neurons; P20‐A, P30‐B: bipolar neurons; P30‐A: a polygonal neuron. Calibration bar: 100 μm. Dors, dorsal; Ant, anterior; Ax, axon; wm, white matter; VI, sixth layer of the cerebral cortex

**Figure 9:**
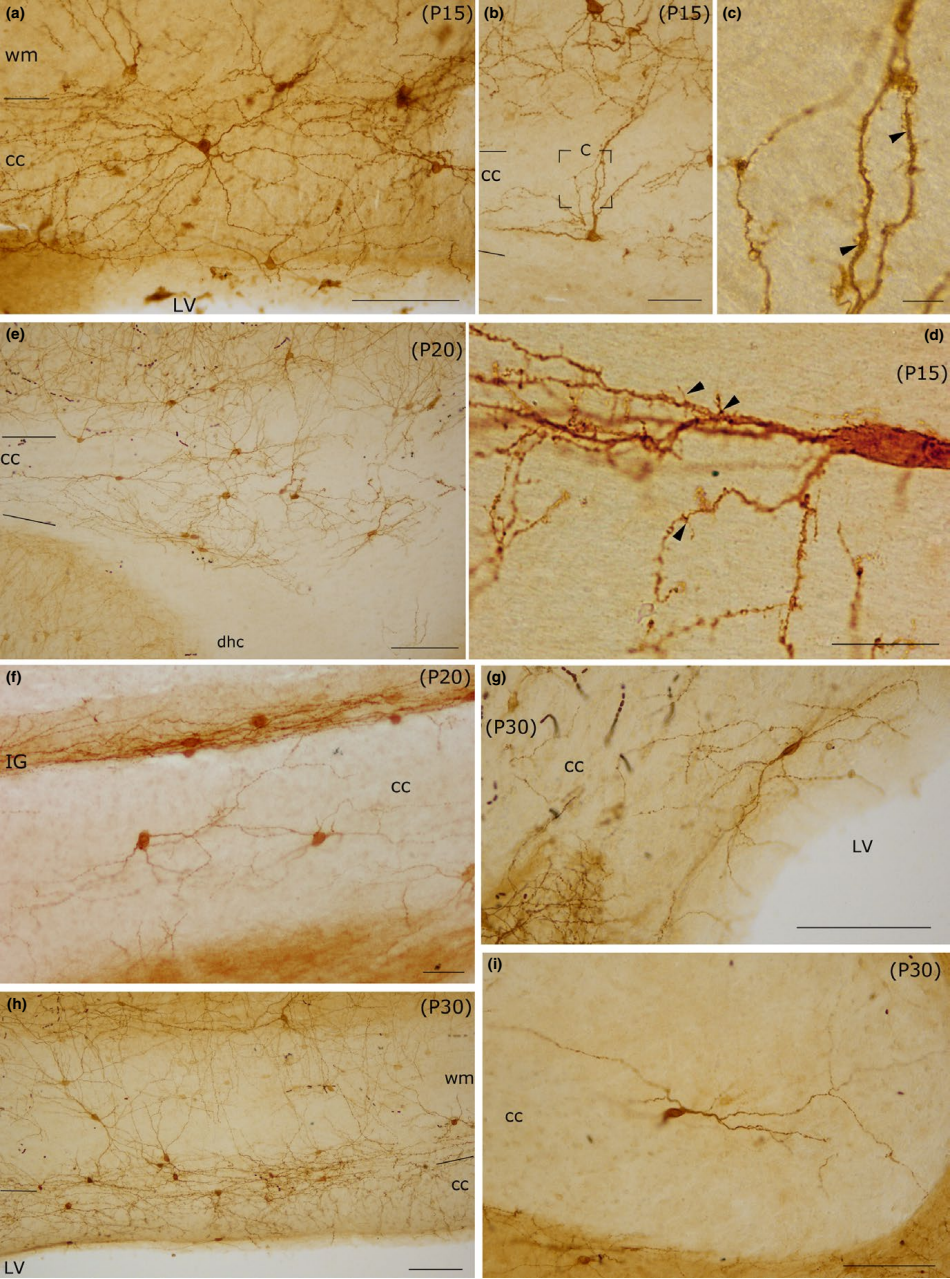
Photomicrographs of intracallosal NK1_IP__‐n_ at different postnatal ages (P15, P20, P30). (a) A round intracallosal NK1_IP__‐n_ whose dendrites are oriented in all directions (P15). (b) A triangular neuron; the apical dendrite crosses the white matter and reaches the cerebral cortex (P15). The framed area, enlarged in c, shows both dendritic appendages and dendritic spines (arrowheads). (d) A bipolar neuron bearing several dendritic appendages (arrowheads) and dendritic spines (P15). Coronal section. (e) Cluster of NK1_IP__‐n_ in the splenium (P20). (f) Two NK1_IP__‐n_ in the medial cc (P20). Some NK1_IP__‐n_ are in the IG, over the cc. (g) A bipolar NK1_IP__‐n_ close to the ependymal region of the cc (P30). (h) Several NK1_IP__‐n_ in the middle cc region (P30). (i) A probable bipolar NK1_IP__‐n_ in the splenium (P30). Calibration bars: 500 μm in a, b, and h; 250 μm in e, g, and i; 100 μm in f; 10 μm in c and d

Intracallosal NK1_IP‐n_ exhibited a further increase in soma size (214.59 μm^2^; Figure 4d), dendritic branching, and dendrite length. Principal and secondary dendrites acquired a dense cover of short and fine appendages (Figure 9d); a large number of typical spines were also interspersed among them (Figures 5, P10‐A, ‐B, ‐C, 6g, 8P15‐A, ‐B and 9c,d). Additionally, short and long thin processes were detected on the somata (Figures 8, P15‐A, ‐B, and 9d). A dense dendrite network was often noted in the thickness of the cc; as in the adult, the network was formed by dendrites of neighboring cells and likely by dendrites of distant neurons that could not be followed to their perikaryon. Several dendrites could be followed both to the ependymal layer and to the overlying white matter. During this stage, many NK1_IP‐n_ were noted in the ependymal region of the cc (Figures 6j and 9a). The number of NK1_IP‐n_ peaked at P10, then slightly declined from P10 to P15 (Figure 3). Their distribution was similar to that of the adult. NK1_IP‐n_ were seen along the rostrocaudal extension of the cc, but showed differences along its lateromedial dimension, being more numerous at the lateral and intermediate levels (Figures 2 and 7b,c).

#### 3.1.5 Postnatal days 15 (P15) to postnatal days 20 (P20)

A definitive maturation of NK1_IP‐n_ occurs at this time. Soma size increased further to 242.32 μm^2^ (Figure 4e); dendrite and soma morphology closely resembled those of the adult; the dendrites could often be followed as far as the overlying white matter (Figure 8, P20‐A). NK1_IP‐n_ were more numerous at the lateral and intermediate levels of the cc (Figures 2 and 7d) and their number showed a further slight reduction (Figure 3).

#### 3.1.6 Postnatal day 20 (P20) to postnatal day 30 (P30)

As reported in a recent study (Barbaresi et al., 2015), intracallosal NK1_IP‐n_ were found throughout the rostrocaudal extension of the cc, but showed a different lateromedial distribution, their number increasing from lateral (stereotaxic plane lat 3.9) to intermediate (stereotaxic plane lat 2.4) and declining at the midline levels (from lat 1.4 to 0.4; Figures 2 and 7e). At P30, their soma measured 262.07 μm^2^ (Figure 4f). At this stage they appeared qualitatively indistinguishable from neurons of adult animals (Barbaresi et al., 2015) and could therefore be classified as bipolar (fusiform and rectangular; Figures 8, P30‐B, and 9g,i), round–polygonal (Figures 8, P20‐B, P30‐A, and 9f,h), and pyramidal (triangular–pyriform; Figure 9f,h). Their number underwent a further slight reduction (Figures 3 and 7e). The dendrites could be followed up for hundreds of microns and bore several swellings and some spines along their course (Figures 8 and 9). Although dendritic spines were not counted, they seemed to be less numerous than those of P 15 rats (Figures 8 and 9). NK1_IP‐n_ dendrites formed a dense network along the rostrocaudal extension of the cc (Figure 9e,h). The dendrites could often be followed as far as the overlying white matter (Figures 8, P20‐A, P30‐B, and 9h).

## 4 Discussion

This study examined the distribution of rat intracallosal neurons expressing SP receptor NK1 on the cellular membrane at different postnatal ages, from birth to postnatal day 30. NK1_IP‐n_ were absent at P0, they increased between P5 and P10, and decreased between P10 and P30.

These findings are consistent with earlier immunocytochemical, autoradiographic, and PCR studies which showed that the expression of rat NK1 receptor is generally higher in the first few days of postnatal life and decreases with aging, although regional variability in NK1 ontogeny has been described.

In the rat trigeminal motor nucleus, NK1 receptor expression peaks at P7 and then declines (Tanaka‐Gomi et al., 2007). Immunocytochemical and western blot analyses indicate that its expression in the hypoglossal nucleus also decrease postnatally (Adachi, Huxtable, Fang, & Funk, 2010). In a RT‐PCR study, Taoka et al. (1996) documented a transiently high level of NK1‐IR mRNA between days 0 and 3, followed by a gradual reduction, in the rat cerebral cortex, hippocampus, and cerebellum. In the striatum, SP receptor binding sites have been seen to form dense patches between P1 and P7 and to decrease thereafter, whereas high densities of binding sites has been reported in most brain stem nuclei of neonatal but not adult rat (Quirion & Dam, 1986).

Charlton and Helke (1986) have described high densities of SP receptor binding sites in the nucleus ventrolateralis and in the intermediolateral cell column of the rat spinal cord since the first postnatal day; those in the phrenic motor nucleus and in the outer laminae of the dorsal horn were not identifiable until after the eighth postnatal day. Also in these nuclei, the net quantity of SP receptor binding sites decreased as the rats aged. According to the authors, the reduction in SP receptor binding sites is not due to the size increase of the spinal cord.

These data also suggest that, in the cc the reduction in intracallosal NK1_IP‐n_ is not due to a dilution related to the cc volumetric expansion, but due to a reduced ability of intracallosal neurons to express the SP receptor.

A key finding was the lack of intracallosal NK1_IP‐n_ at P0 and their progressive increase in size and number during postnatal development. The absence of intracallosal NK1_IP‐n_ at P0 seems to be specific for two reasons: (1) in the same sections, dense labeling was observed in the medulla oblongata, caudate putamen, hippocampus, and cerebral cortex, in accordance with previous immunocytochemical, HPLC–radioimmunoassay, and autoradiographic studies (Ardelt, Karpitskiy, Krause, & Roth, 1996; Diez‐Guerra, Veira, Augood, & Emson, 1989; Horie et al., 2000; Mensà, 2013; Quirion & Dam, 1986); (2) P0 sections were processed with those from P30 animals, where labeling was similar to that reported in a previous study (Barbaresi et al., 2015). However, these findings do not rule out the possibility that NK1 expression in intracallosal neurons at P0 was below the detection threshold of the immunocytochemical techniques used in the study.

### 4.1 Comparison with other studies

The progressive increase in the number of intracallosal NK1_IP‐n_ seen at different postnatal ages, especially from P0 to P5 and from P5 to P10, and the large number of neurons found in the adult (Barbaresi et al., 2015), contrast with most previous studies, since a prominent feature of cc development is the presence of transient neurons and fibers (Innocenti, 1986). A transient GABAergic neuronal population migrates tangentially to invade the rat cc in late prenatal and early postnatal life (DeDiego, Smith‐Fernández, & Fairén, 1994) and gradually decreases in the subsequent postnatal stages. Another population of intracallosal neurons in the developing rat cc, NPY‐positive cells, increases up to P7 (Ding & Elberger, 2000) and then gradually decreases to become very rare in the adult cc (Ding & Elberger, 2000; Woodhams et al., 1985). Reelin‐expressing cells are seen in the rat and mouse cc at P7, P14, and P21 (Misaki, Kikkawa, & Terashima, 2004); however, since the authors provide no information either on the number of intracallosal neurons found at the various postnatal ages or on the number found in the adult, these data are difficult to evaluate and to compare with those found in the present study.

A recent immunocytochemical study has shown that chain migrating interneurons positive for Sp8 (a transcription factor) transiently cross the cc during the second postnatal week (Cai, She, & Wang, 2015).

MAP2 immunopositive neurons also appear transiently in the cat cc during postnatal development. At birth, they are about 570, they then drop to about 200 in the adult (Riederer et al., 2004). Similar data have been documented for intracallosal neurons in the human cc. Intracallosal neurons labeled with neuronal markers (MAP2, NeuN, NPY, calretinin, calbindin) were more numerous and more morphologically complex at the end of the fetal period, and decreased after the first postnatal year; only 5–10% of the initial intracallosal neuronal population remained in the adult (Jovanov‐Milošević et al., 2010).

Moreover, GABA‐like immunoreactive (ir) axons have been reported in the rat cc until postnatal day 6; they were grouped in dense bundles and most of them disappeared in the older rats (Cobas, Alvarez‐Bolado, & Fairén, 1988). Very few and sparse GABA‐containing fibers persist in the adult rat cc (Ottersen & Storm‐Mathisen, 1984). These fibers could be axons of cortical cells projecting transiently through the cc. In an in vivo study performed in rat pups (from P0 to P1), combining retrograde labeling with electrophysiology and immunocytochemistry, Kimura and Baughman (1997) found a population of GABAergic callosal neurons accounting for between 21% and 57% of the whole callosal population. In the adult, GABAergic callosal neurons are reduced to 0.7–1% of the whole callosal population (Fabri & Manzoni, 2004; Gonchar et al., 1995).

The developing rat cc also contains a transient population of NPY_ir_ and SOM_ir_ fibers. Both fiber populations initially increase, they peak at P10, then decrease to mature levels. Only few NPY_ir_ and SOM_ir_ axons are found in the adult cc (Ding & Elberger, 2000). These fibers could be axons sent through the cc to the contralateral hemisphere by transitory NPY_ir_ and SOM_ir_ neuronal populations found in the rat cerebral cortex (Ding & Elberger, 1994, 2000).

The second important finding of our study regards the distribution of intracallosal NK1_IP‐n_ during postnatal development. At variance with earlier reports, we found that from P5 onward their distribution was similar to that described in the adult cc (Barbaresi et al., 2015), in that they were more numerous in the lateral cc and gradually decreased approaching the midline, where they were few or absent; in contrast, in cat and human cc, the distribution of intracallosal neurons in early postnatal development is different to that of the adult cc.

In neonatal cats, MAP2‐positive neurons are found throughout the cc; at later postnatal ages they are confined to the cc boundaries, and in the adult they are found only in the ventral area of the rostrum (Riederer et al., 2004). Similar results have been reported in humans, where in the perinatal period NeuN immunopositive intracallosal neurons have been detected in the dorsal cc, whereas in the adult they were found both in the rostrum and in the genu (Jovanov‐Milošević et al., 2010).

### 4.2 Hypothesis on the functional role of the intracallosal NK1_IP‐n_

The presence and numerical growth of intracallosal NK1_IP‐n_ between P5 and P30 could be related to the myelination process of cc axons. A recent double‐labeling immunofluorescent study has demonstrated that in the adult rat cc nearly all intracallosal NK1_IP‐n_ colocalize with neuronal nitric oxide synthase (nNOS), the enzyme that synthesizes NO (Barbaresi et al., 2015). It may thus be hypothesized that NK1_IP‐n_ containing nNOS could be present even in the cc of younger animals. This hypothesis is supported by the increase in NO‐producing neurons in the deep white matter of the rat cerebral cortex during postnatal development (Clancy, Silva‐Filho, & Friedlander, 2001). The excitation of these neurons by SP (via synaptic contact or volume transmission) could lead to NO production through different second messenger systems (Bredt & Snyder, 1990; Khawaja & Rogers, 1996; Quartara & Maggi, 1997; Saria, 1999; Vincent, 1994) subsequently to its release. In turn, released NO would stimulate the growth and differentiation of oligodendrocytes, which are responsible for the myelination of callosal axons (Garthwaite, Hampden‐Smith, Wilson, Goodwin, & Garthwaite, 2015; Tanaka, Markerink‐Van Ittersum, Steinbush, & De Vente, 1997) that occurs during the first month of life (Seggie & Berry, 1972; Valentino & Jones, 1982). The importance of NO in cc myelination processes is also demonstrated by other studies. nNOS‐deficient mice show a delay in remyelination following chemical demyelination (Liñares et al., 2006), and Sprague Dawley pups inhaling NO‐enriched air during first postnatal week show increased myelination of cc axons (Olivier et al., 2010).

NO exerts a variety of effects on axons. As in the retinotectal system, NO may be required for pathfinding and may play an important role in neuronal growth cone morphogenesis, axonal guidance (Berman & Morris, 2011; Cossenza et al., 2014; Nikonenko, Jourdain, & Muller, 2003; Williams, Nordquist, & McLoon, 1994), and the refinement processes (Ernst, Jurney, & McLoon, 1998; Wu, Williams, & McLoon, 1994) that involve multiple complex mechanisms and a variety of molecules besides NO (Kalil, Li, & Hutchins, 2011; Niquille et al., 2009; Tessier‐Lavigne & Goodman, 1996).

As in the adult (Barbaresi et al., 2015), NO released from intracallosal NK1‐expressing neurons may also be involved in cerebrovascular control mechanisms (Iadecola, 2004) or in modulating arterial blood flow during cerebral ischemia in rat pups (Bonnin et al., 2012).

In parallel with their number, the size of intracallosal NK1_IP‐n_ also underwent a considerable increase. Since measurements were not made in the plane of the nucleolus (Anamizu, Seichi, Tsuzuki, & Nakamura, 2006; Offord, Ota, Oenning, & Dyck, 1974), their actual size could not be measured; however, our experimental approach allowed documenting a steady size increase of intracallosal NK1_IP‐n_ in the first four postnatal weeks, consistent with reports in the overlying cerebral cortex, where the growth of projecting (pyramidal) and local circuit neurons accelerates in the first 3 weeks of postnatal development to achieve adult size at the end of the fourth week (Miller, 1984). Between P5 and P10, the dendritic tree of NK1_IP‐n_ increased gradually, as described in cerebral cortex neurons (Miller, 1984). Since the dendrites NK1_IP‐n_ extend along the anteroposterior axis of the cc and toward the overlying cerebral cortex, it is possible for intracallosal NK1_IP‐n_ to be activated by SPergic elements of the cerebral cortex. At this time of postnatal development, individual SP_ir_ neurons issue axons in the sixth layer of the cerebral cortex and even in the white matter (Del Rio, Soriano, & Ferrer, 1991) that potentially could innervate intracallosal NK1_IP‐n_ dendrites. Another possibility is that SP released from these cortical neurons could act on intracallosal NK1_IP‐n_ in a paracrine‐like manner, since SP can diffuse across a significant distance from its site of release to bind to a receptor (Liu et al., 1994; Nakaya et al., 1994; Vruwink, Schmidt, Weinberg, & Burette, 2001; Wolansky, Pagliardini, Greer, & Dickson, 2007). In addition, the present data showed that from P10 onward neurons were more often grouped in clusters, with their dendrites forming a dense network close to the ependymal layer or to the middle and dorsal cc. Even more frequently, NK1_IP‐n_ were found close to the ependymal layer; this suggests that they may be in contact with cerebrospinal fluid (CSF) through their dendrites, axons, or perikarya, and that they may belong to the CSF‐contacting neuronal system found in many vertebrate periventricular brain regions (Vigh et al., 2004). Although CSF contains a relatively high amount of SP in the adult brain (Muñoz & Coveñas, 2014), little is known about its SP concentration in early stages of the rat cerebral development. High levels of SP have been reported in CSF of fetuses and children (Tam, Dockray, & Lister, 1985). If this also applies to the rat, then ependymal NK1_IP‐n_ could be activated, more intensely than in adults, via volume transmission by diffusion of SP from CSF through the intercellular space (Abbadie, Skinner, Mitrovic, & Basbaum, 1999; Barbaresi et al., 2015; Ramer, 2008), thus playing an important role in neurodevelopment processes as suggested for humans (Tam et al., 1985). Such neurons could also be involved in CSF composition and in the regulation of its pH and osmolality (Vigh et al., 2004).

An important and intriguing feature of intracallosal NK1_IP‐n_, found in the present study, is the presence of dendritic filopodia mixed with dendritic spines, which seemed to be particularly numerous between P10 and P15 and then declined during the postnatal development. What is the function of these dendritic protrusions? Although there is evidence that both dendritic filopodia and spines are involved in synaptogenesis during development of several of CNS regions (Ziv & Smith, 1996), their functional role in the cc is less clear. Moreover, an exuberant and transient projection, formed by an excess of callosal projecting neurons and callosal axon branching, has been described in the cc during the first stage of development (Innocenti, 1986; Kadhim, Bhide, & Frost, 1993; O'Leary, Stanfield, & Cowan, 1981). Are these spines a target for transient axonal branching of permanent callosal axons? The findings described above suggest that, during development, dendritic protrusions grow and search for nearby axons to synapse with; thereafter they disappear due, for example, to retraction of transient axonal branching of permanent callosal axons (Kadhim et al., 1993). In support for this hypothesis, a similar sequence of events seems to take place in the development of the afferent innervation of other CNS regions (Ramoa, Campbell, & Shatz, 1988; Saito et al., 1992).

According to our findings, dendritic filopodia decreased as age increased, and only spines were found on NK1 dendrites at P20 and P30. Dendritic spines are sites of excitatory synaptic transmission, and their structure and density are important measures of synaptic function (Pannese, 1994; Peters, Palay, & Webster, 1991). It is therefore likely that intracallosal NK1_IP‐n_ receive some synaptic contact. The hypothesis is supported by an early electron microscopic study describing synapses on intracallosal neurons (Ling & Ahmed, 1974). Moreover, NK1_IP‐n_ receiving neurochemically diverse synaptic inputs have been described in other fibrous tracts such as the dorsal columns of several mammals (Abbadie et al., 1999; Ramer, 2008).

## 5 Conclusion

The main findings of this study may be summarized as follows: (1) intracallosal neurons expressing NK1, the principal SP receptor, are visible since P5; (2) at P5, their distribution is already similar to that seen in the adult; (3) their number increase between P5 and P10 then declines, but unlike other intracallosal neurons, NK1_IP‐n_ make up a significant population in the adult cc; (4) intracallosal NK1_IP‐n_ size increase with age; (5) starting at P20, intracallosal NK1_IP‐n_ form a heterogeneous population. These neurons may act as temporary targets for ingrowing callosal axons, take part in the mechanism of axonal myelination of callosal fibers, and play an important role in callosal pathfinding. Since they are also found in adult rat cc, it is likely that their role changes during lifetime (Friedlander & Torres‐Reveron, 2009; Rockland & Nayyar, 2012).

## Conflicts of Interest

None declared.
